# Dissociable neural correlates of multisensory coherence and selective attention

**DOI:** 10.1523/JNEUROSCI.1310-22.2023

**Published:** 2023-05-19

**Authors:** Fei Peng, Jennifer K. Bizley, Jan W. Schnupp, Ryszard Auksztulewicz

**Affiliations:** 1 Department of Neuroscience, City University of Hong Kong, Hong Kong, China; 2 Ear Institute, University College London, London, United Kingdom; 3 Center for Cognitive Neuroscience Berlin, Department of Education and Psychology, Free University Berlin, Germany

**Keywords:** temporal coherence, selective auditory attention, audio-visual binding, object formation

## Abstract

Previous work has demonstrated that performance in an auditory selective attention task can be enhanced or impaired, depending on whether a task-irrelevant visual stimulus is temporally coherent with a target auditory stream or with a competing distractor. However, it remains unclear how audiovisual (AV) temporal coherence and auditory selective attention interact at the neurophysiological level. Here, we measured neural activity using electroencephalography (EEG) while human participants (men and women) performed an auditory selective attention task, detecting deviants in a target audio stream. The amplitude envelope of the two competing auditory streams changed independently, while the radius of a visual disc was manipulated to control the audiovisual coherence. Analysis of the neural responses to the sound envelope demonstrated that auditory responses were enhanced independently of the attentional condition: both target and masker stream responses were enhanced when temporally coherent with the visual stimulus. In contrast, attention enhanced the event-related response (ERP) evoked by the transient deviants, independently of AV coherence. Finally, in an exploratory analysis, we identified a spatiotemporal component of ERP, in which temporal coherence enhanced the deviant-evoked responses only in the unattended stream. These results provide evidence for dissociable neural signatures of bottom-up (coherence) and top-down (attention) effects in AV object formation.

## Introduction

In many real world sound environments, sounds originate from multiple sources – the auditory system needs to appropriately segregate and group sound features to efficiently process the entire scene (Maddox & Shinn-Cunningham, 2012; Middlebrooks et al., 2017; Shamma et al., 2011). Several psychoacoustic studies have demonstrated that visual cues which are temporally coherent with sounds can modulate auditory processing. For example, a synchronous, task-irrelevant light flash improves the detection of weak auditory signals (Lovelace et al., 2003). Similarly, task-irrelevant visual stimuli which are temporally coherent with a speech envelope enhance speech intelligibility in background babble noise (Yuan et al., 2020). Furthermore, performance in an auditory selective attention task can be enhanced or impaired, depending on whether the task-irrelevant visual stimulus is temporally coherent with a target sound stream or a competing masker stream (Maddox et al., 2015). However, the neural mechanisms mediating the interactions between temporal coherence and selective attention in facilitating AV integration remain unknown.

Several previous studies have identified potential neural correlates of attentional modulation of AV integration. For example, a study using simple tone pips and visual gratings demonstrated that ERPs related to multisensory integration were amplified by selective attention (Talsma & Woldorff, 2005). When both visual and auditory stimuli were attended, the ERP peak amplitude showed superadditive AV effects; however, subadditive effects were observed for unattended stimuli (Talsma et al., 2007). Some EEG and MEG studies have employed the analysis of “neural envelope-tracking responses” to speech, by modeling the relationship between neural activity and the auditory envelope (Crosse et al., 2015; Golumbic et al., 2013), and have found that congruent audio-visual speech enhances the envelope tracking response relative to auditory speech alone or the linear summation of auditory and visual speech. Other studies have used auditory selective attention tasks to show that attention is necessary for AV speech integration. For example, Morís Fernández et al. (2015) measured fMRI data and showed that multisensory integration occurred almost exclusively only when the congruent AV speech was attended. However, Ahmed et al., (2021) measured EEG and found some evidence for early AV integration in the unattended stream, consistent with the idea that distinct audiovisual computations emerge at different processing stages (Kayser & Shams, 2015; Talsma et al., 2007; Talsma & Woldorff, 2005; Zumer et al., 2020). One potential difficulty with interpreting findings from AV speech processing is that it can be hard to know the extent to which they generalize to other continuous AV stimuli, given that speech processing can be heavily influenced by linguistic knowledge and expectations. Thus these speech specific studies might not represent more general mechanisms of visual influences on auditory processing. Consistent with AV integration occurring independently of attention for non-speech stimuli, neural correlates of AV integration were observed in single neurons in the auditory cortex of passively exposed ferrets. This included enhancement of the neural representation of the temporally coherent features (i.e., envelope), but also of the other (i.e., timbre) sound features (Atilgan et al., 2018). Together, from these findings it remains unclear whether such bottom-up effects modulate the cortical representation of auditory streams independently of attentional top-down enhancement, or whether these effects are synergistic.

Here, we use EEG to investigate the electrophysiological correlates of AV temporal coherence and auditory selective attention on sound processing in an auditory selective attention task. Listeners were required to detect short timbre deviants in an attended audio stream, while a visual stimulus was paired with either the target, masker or neither sound through coherent size/amplitude fluctuations. First, we focused our analysis on how AV coherence and attention affected the neural signatures of continuous stream processing, as manifest in the envelope-tracking response. Second, we focused on the transient auditory deviants, whose timing was independent of the features of the visual stream, and compared deviant-evoked ERPs between conditions. Our goal was to test the hypothesis that attention and audiovisual integration operate independently.

## Materials and Methods

### Participants

Twenty volunteers were recruited for this experiment (median ± standard deviation (SD) age, 22 ± 2 years; 12 males; 19 right-handed). All participants were healthy, had self-reported normal hearing and normal or corrected-to-normal vision. Prior to the experiment, each participant gave written informed consent. All procedures were approved by the Human Subjects Ethics Sub-Committee of the City University of Hong Kong.

### Stimuli

**Figure 1:**
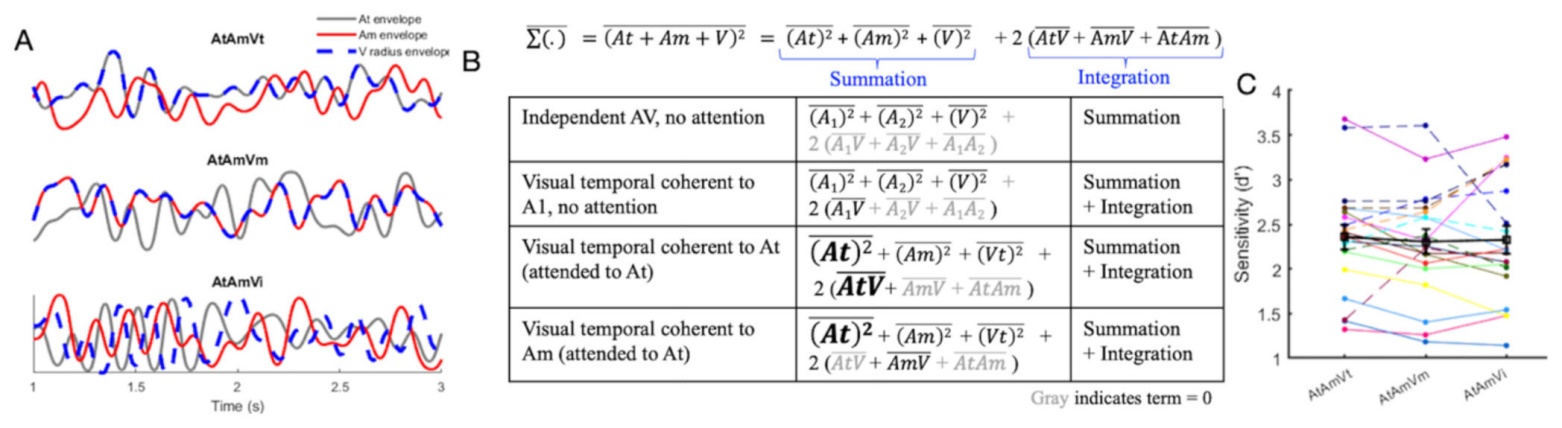
Experimental paradigm, diagram of the possible effects of attention and coherence, and behavioral performance. **(A)** Schematic plot of auditory and visual stimuli in the behavioral task. Amplitude envelopes of target/attended sound (grey solid line), masker/unattended sound (red solid line), and visual radius envelope (blue dashed line). **(B)** Diagram of the possible effects of attention and coherence. Attention and coherence effects can be mapped onto four main scenarios: for non-coherent stimuli in the absence of selective attention, the response is a linear summation of the three streams; for coherent stimuli, there is an additional term representing audiovisual integration. In the context of a task requiring selective attention to one sound, attention can enhance (illustrated by larger terms) either the auditory stream only (for incoherent stimuli) or additionally the integration term (for coherent stimuli). This model assumes that when temporal coherence is absent the relevant integration term becomes zero, and these are shown in gray. **(C)** Behavioral sensitivity (d’) for each visual condition. Each line shows data of one participant. Solid lines indicate participants with higher d’ in the AtAmVt vs. AtAmVm condition, and dashed lines indicate participants with lower d’ in the AtAmVt vs. AtAmVm condition. Black squares represent group averages, and error bars indicate the standard error of the mean (SEM).

We adapted the behavioral paradigm from previous psychoacoustics studies (Atilgan & Bizley, 2021; Maddox et al., 2015). Stimuli included two simultaneously presented auditory streams and one visual stream. One auditory stream was meant to be attended, and will be referred to as the target sound (At), while the other one was meant to be unattended, and will be referred to as the masker stream (Am). Finally, stimulation included a concurrently presented visual stream (V) which comprised a radius-modulated disc. Auditory streams were independently amplitude-modulated and the modulation of the visual disc could be temporally coherent either with the amplitude of the target stream (AtAmVt), the masker stream (AtAmVm), or independent of both (AtAmVi) (Figure 1A).

The envelopes below 7 Hz were generated using the same methods as in Maddox et al. (2015). Briefly, frequency domain synthesis was used to generate the envelopes. In the frequency domain, amplitudes of frequency bins between 0-7 Hz were set to one and, for other frequency bins, to zero. The non-zero bins were given a random phase from a uniform distribution between 0 and 2π, at an audio sampling rate of 24414 Hz. To maintain Hermitian symmetry, the corresponding frequency bins across Nyquist frequency were set to the respective complex conjugates. The inverse Fourier transform was calculated to create the time domain envelope. Three envelopes of each trial were computed using the same method, and they were orthogonalized using the Gram-Schmidt procedure. Visual envelopes were generated by downsampling the auditory envelope at the monitor frame-rate of 60 Hz, where the disc radius of the first frame was corresponding to the first auditory sample. Each auditory stream consisted of one continuous amplitude modulated synthetic vowel, either /u/ or /a/, which were generated by filtering a click train at four “formant” frequencies (F1-F4). The fundamental frequency (F0) of vowel /u/ was 175 Hz, and the formant peaks were 460, 1105, 2975, 4263 Hz, while the F0 of vowel /a/ was 195 Hz, and the formant peaks were 936, 1551, 2975, 4263 Hz. Auditory deviants were embedded in the auditory streams by temporarily changing the timbre of the vowel. The deviant in vowel /u/ transitioned (in F1/F2 space) towards the vowel /ε/, with the maximum timbre change resulting in formant peaks at 525, 1334, 2975, 4263 Hz, while the deviant in vowel /a/ transitioned towards /i/ with formant peaks at 860, 1725, 2975, 4263 Hz. The duration of each deviant was 200 ms, which included a linear change of the formants towards the deviant for 100 ms and then back for 100 ms.

The visual stimulus was a grey disc surrounded by a white ring presented at the center of the black screen. The radius of the visual stimulus was modulated by the visual envelope, such that the disc subtended between 1° and 2.5°, and the white ring extended 0.125° beyond the grey disc.

Each trial lasted 14 s and comprised three streams. A target audio stream and the visual stream were each 14 s in duration while the masker stream, although also generated to be 14 s in duration, was silenced for the first second. The initial 1 s, during which only the target stream was audible, provided the cue for the listener which was the to-be-attended target stream. Auditory deviants could occur at any time during a window beginning 2 s after the onset of the target audio stream and ending 1 s before the end of the trial, subject to the constraint that the minimum interval between auditory deviants was 1.2 s. On average each stream contained 2 deviants (range 1-3 across trials). Unlike Maddox et al. (2015), the visual stream did not contain any colour deviants.

### Procedure

Participants were seated in a sound-attenuated room. Auditory stimuli were presented binaurally via earphones (ER-3, Etymotic Research, Elk Grove Village, IL, USA), using an RZ6 signal processor at a sampling rate of 24414 Hz (Tucker-Davis Technologies, Alachua, FL, USA). The sound level was calibrated at 65 dB SPL. Visual stimuli were presented on a 24-inch computer monitor using the Psychophysics Toolbox for MATLAB. Participants were asked to pay attention to the target auditory stream and to detect the embedded auditory deviants by pressing a keyboard button. They were instructed to refrain from pressing buttons in response to any events in the masker stream.

Before the actual task, all participants completed a training session to verify that they were able to detect the auditory deviants. The training session included four blocks, and each block included 9 trials. The feedback of performance was given after each block, and all participants showed they could perform the experiment (d’ > 0.8) in at least one block of four.

Participants were instructed to minimize eye blinks and body movements during the EEG recording. Continuous EEG signals were collected using an ANT Neuro EEGo Sports amplifier from 64 scalp channels at a sampling rate of 1024 Hz. The EEG signals were grounded at the nasion and referenced to the Cpz electrode. Each participant completed 12 blocks of the task, with 18 trials (6 trials x 3 conditions) in each block. Trials belonging to different conditions were presented in a randomly interleaved order. In total, each participant completed 216 trials (72 trials x 3 conditions). Feedback on behavioral performance was provided after each block. Triggers corresponding to trial and deviant onset were recorded along with the EEG signal.

### Behavioral data analysis

A ‘hit’ was defined as the response to the deviant in the target auditory stream within 1 s following the onset of the deviant, and a ‘false alarm’ was defined as the response to a deviant that occurred in the masker stream. To study how visual coherence affects auditory deviant detection, we conducted a one-way repeated measures ANOVA on the sensitivity measure d’ with a within-subjects factor of AV condition (visual coherent with the target, AtAmVt, visual coherent with the masker, AtAmVm, and independent visual AtAmVi).

### EEG signal pre-processing

EEG signals were pre-processed using the SPM12 Toolbox (Wellcome Trust Centre for Neuroimaging, University College London) for MATLAB. Continuous data were downsampled to 500 Hz, high-pass filtered at a cut-off frequency of 0.01 Hz, notch-filtered between 48 Hz and 52 Hz, and then low-pass filtered at 90 Hz. All filters were fifth-order zero-phase Butterworth. Eyeblink artifacts were removed by the use of the principal component analysis (PCA) based on a “preselection” spatial filtering technique described by Ille et al., (2002). Specifically, eyeblink artifacts were detected by computing the principal components of the signal in the channel Fpz, and removed by subtracting the first two spatiotemporal components associated with each eyeblink from all channels (Ille et al., 2002). The EEG data were then re-referenced to the average of all channels. The preprocessed data were further analyzed in two ways: For the response to the sound amplitude envelope, the pre-processed data were bandpass filtered between 0.3 and 30 Hz (Crosse et al., 2015), downsampled to 64 Hz, and subjected to a calculation of the TRF, or used for stimulus reconstruction (see below). For the deviant evoked response analysis, the pre-processed data were epoched from -100 ms to 500 ms relative to deviant onset. Epoched EEG signals were then denoised using the “Dynamic Separation of Sources” (DSS) algorithm (de Cheveigné & Simon, 2008), which is commonly used to maximize reproducibility of stimulus-evoked response across trials and maintain the differences across the different stimulus types (here: 2 vowel types × 3 experimental conditions). Epoched data were linearly detrended, and the first seven DSS components were preserved and applied to project the data back into sensor space. The SD of the voltage over time was computed for each trial, and we excluded the noisy trials whose SD exceeded the median ± 2SD over trials. Across participants roughly 30 trials were excluded for each participant (the included trials were 829 ± 31 (median ± SD) out of 864 trials). Denoised data were averaged across the good trials.

## EEG response to sound amplitude envelopes

### Stimulus reconstruction

To investigate how visual temporal coherence and attention affect multisensory integration, we quantified the accuracy of neural tracking of the sound amplitude envelope. We reconstructed amplitude envelopes of different elements of the AV scene (Crosse et al., 2015) based on the EEG data using a linear model as follows:

(1) $$\overset{ˇ}{s}(t)=\sum_{n=1}^{64}\sum_{\tau =0}^{500ms}r(t+\tau ,n)g(\tau ,n)$$

where *š*(*t*) is the reconstructed envelope; *r*(*t* + *τ*,*n*) is the EEG data at channel *n*; and *g* is the linear decoder representing the linear mapping from the response to stimulus amplitude envelope at time lag *τ*. The time lag *τ* ranged from 0 to 500 ms post-stimulus. The decoder was obtained separately for each condition using ridge regression as follows:

(2) $$g=(R^{T}R+\lambda I{)}^{-1}R^{T}S$$

where *R* is the lagged time series of the EEG response, *λ* is the ridge parameter, *I* is the regularization term, and *s* is the sound amplitude envelope. The decoder is a multivariate impulse response function computed from all channels and all time-lags simultaneously. Decoders corresponding to the AV, A-only, and V-only streams were generated separately as follows:

(3) $$g_{\text{AtVt}\;}(\tau ,n)=(R_{\text{AtAmVt}\;}^{T}R_{\text{AtAmVt}\;}+\lambda I{)}^{-1}R_{\text{AtAmVt}}^{T}s_{\text{AtVt}\;}$$

(4) $$g_{AmVm}(\tau ,n)=(R_{AtAmVm}^{T}R_{AtAmVm}+\lambda I{)}^{-1}R_{AtAmVm}^{T}s_{AmVm}$$

(5) $$g_{At}(\tau ,n)=(R_{AtAmVi}^{T}R_{AtAmVi}+\lambda I{)}^{-1}R_{AtAmVi}^{T}s_{At}$$

(6) $$g_{Am}(\tau ,n)=(R_{AtAmVi}^{T}R_{AtAmVi}+\lambda I{)}^{-1}R_{AtAmVi}^{T}s_{Am}$$

(7) $$g_{Vi}(\tau ,n)=(R_{AtAmVi}^{T}R_{AtAmVi}+\lambda I{)}^{-1}R_{AtAmVi}^{T}s_{Vi}$$

Since in the condition AtAmVi, the envelope of At, Am, and Vi are independent of each other, we could obtain the decoder of the envelopes of the auditory target only, auditory masker only, and visual only, respectively. To obtain the decoder for each condition, we used leave-one-trial-out cross-validation to select the *λ* value (from the set of 10^-6^, 10^-5^, …, 10^5^, 10^6^) for which the correlation between *š*(*t*) and *s(t)* is maximized. To assess the effect of AV integration, we reconstructed the sound envelopes (both target and masker sound) using the integration AV decoder and the algebraic sum of the A and V decoder (A+V), separately, based on the following formulas:

(8) $$\overset{⌣}{s_{At_{-}(AV)}}(t)=\sum_{n=1}^{64}\sum_{\tau =0}^{500ms}r_{AtAmVt}(t+\tau ,n)g_{AtVt}(\tau ,n)$$

(9) $$\overset{⌣}{s_{At_{-}(A+V)}}(t)=\sum_{n=1}^{64}\sum_{\tau =0}^{500ms}r_{AtAmVt}(t+\tau ,n)\left(g_{At}(\tau ,n)+g_{Vi}(\tau ,n)\right)$$

(10) $$\overset{⌣}{s_{Am_{-}(AV)}}(t)=\sum_{n=1}^{64}\sum_{\tau =0}^{500ms}r_{AtAmVm}(t+\tau ,n)g_{AmVm}(\tau ,n)$$

(11) $$\overset{⌣}{s_{Am_{-}(A+V)}}(t)=\sum_{n=1}^{64}\sum_{\tau =0}^{500ms}r_{AtAmVm}(t+\tau ,n)\left(g_{Am}(\tau ,n)+g_{Vi}(\tau ,n)\right)$$

The reconstruction accuracy (r) was defined as the Pearson correlation coefficient between the actual stimulus envelope and the estimated envelope.

Based on our main research question - namely whether the effects of attention and coherence are independent or synergistic - the possible scenarios of combining the effects of coherence and attention were considered in the context of two main models of AV coherence: an integration model and a summation model (Figure 1B). To test whether the reconstruction accuracy using either the AV decoder (“integration model”) and/or A+V decoder (“summation model”) was significantly larger than chance, we conducted a nonparametric permutation test. The null distribution of 1000 Pearson’s r values was created for each subject by calculating the correlation between randomly shuffled response trials of estimated sound envelopes and actual sound envelopes. We estimated sound envelopes using each decoder separately, and generated the null distribution for each condition.

To test for the interaction of attention and AV integration, we computed a repeated-measures ANOVA on reconstruction accuracy with two main within-subjects factors, attention (target vs. masker) and integration decoder (“integration model”: AV vs. “summation model”: A+V).

### Temporal response function (TRF) estimation

To investigate how the visual temporal coherence and attention affect AV integration across the EEG channels, we estimated the linear temporal response function (TRF) (Crosse, Di Liberto, Bednar, et al., 2016) which links the EEG response at each channel and the sound envelope. The TRF is the linear filter that best describes the brain’s transformation of the sound envelope to the continuous neural response at each EEG channel location (Haufe et al., 2014). TRFs were estimated separately for each experimental condition (AtAmVt, AtAmVm, AtAmVi) as follows:

(12) $$r_{AtAmVt}(t,n)=\sum_{\tau }s_{AtVt}(t-\tau )TRF_{AtVt}(\tau ,n)+\sum_{\tau }s_{Am}(t-\tau )TRF_{Am1}(\tau ,n)+\varepsilon (t,n)$$

(13) $$r_{AtAmVm}(t,n)=\sum_{\tau }s_{At}(t-\tau )TRF_{At1}(\tau ,n)+\sum_{\tau }S_{AmVm}(t-\tau )TRF_{AmVm}(\tau ,n)+\varepsilon (t,n)$$

(14) $$r_{AtAmVi}(t,n)=\sum_{\tau }s_{At}(t-\tau )TRF_{At}(\tau ,n)+\sum_{\tau }s_{Am}(t-\tau )TRF_{Am}(\tau ,n)+\sum_{\tau }S_{Vi}(t-\tau )TRF_{Vi}(\tau ,n)+\varepsilon (t,n)$$

where *r_AtAmVt_*, *r_AtAmVm_*, and *r_AtAmVi_* are the EEG response in each of the 3 conditions respectively; *t* is time, *n* is the index of the EEG channel under consideration; *s_At_*, *s_Am_,* and *s_Vi_* are the stimulus envelopes of At, Am, and Vi, respectively; *τ* represents the convolution time lag (-100 ms to 500 ms), and *ε*(*t*, *n*) is the residual “error”, that is, the part of the EEG recording not explained by the TRF model. We use the term *TRF_At1_* to describe the TRF in the AtAmVm condition, and *TRF_Am1_* in the AtAmVt condition, to differentiate them from the *TRF_At_* and *TRF_Am_* estimated from the AtAmVi condition, this being the only condition in which all three streams were fully independent (Equations 13–15). The TRF for each condition was calculated at time lags from -100 ms to 500 ms relative to the stimulus as follows:

(15) $$TRF_{\text{AtVt}\;}=(S_{\text{AtVt}\;}^{T}S_{\text{AtVt}\;}+\lambda I{)}^{-1}S_{AtVt}^{T}r_{AtAmVt}$$

(16) $$TRF_{AmVm}=(S_{AmVm}^{T}S_{AmVm}+\lambda I{)}^{-1}S_{AmVm}^{T}r_{AtAmVm}$$

(17) $$TRF_{At}=(S_{At}^{T}S_{At}+\lambda I{)}^{-1}S_{At}^{T}r_{AtAmVi}$$

(18) $$TRF_{Am}=(S_{Am}^{T}S_{Am}+\lambda I{)}^{-1}S_{Am}^{T}r_{AtAmVi}$$

(19) $$TRF_{Vi}=(S_{Vi}^{T}S_{Vi}+\lambda I{)}^{-1}S_{Vi}^{T}r_{AtAmVi}$$

where ***S*** is the lagged time series of the stimulus envelope; *I* is the regularization term used to prevent overfitting; and *λ* is the ridge parameter. *TRF_Atvt_*, *TRF_AmVm_, TRF_At_*, *TRF_Am_*, and *TRF_vi_* were fitted separately for each condition using the MATLAB toolbox adapted from a previous study by Crosse et al. (2016). The TRF of each channel was estimated using leave-one-out crossvalidation. The best *λ* (in the range of 2^10^, 2^11^, …, 2^21^) was selected based on the maximum correlation coefficient between the predicted response with the actual neural response for each channel. The EEG signal of each trial (13 s long) was used to estimate the TRF, modeling the neural response to the simultaneous presentation of both At and Am.

To test whether AV integration is affected by attention, we compared the TRF amplitude between the temporally coherent and independent conditions across EEG channels and time points. Single-participant TRF data were converted into three-dimensional images (2D: spatial topography, 1D: time) and entered into a repeated-measures ANOVA with two within-subjects factors: attention (attended: *TRF_AtVt_* and *TRF_At_* + *TRF_Vi_* , unattended: *TRF_AmVm_* and *TRF_Am_* + *TRF_Vi_*) and integration (integration model: *TRF_AtVt_* and *TRF_AmVm_,* linear summation model: *TRF_At_* + *TRF_Vi_* and *TRF_Am_* + *TRF_Vi_*). The two-way repeated-measures ANOVA was implemented as a GLM in SPM12. The resulting statistical parametric maps, representing the main and interaction effects, were thresholded at p < 0.05 (two tailed) and corrected for multiple comparisons across spatiotemporal voxels at a family-wise error (FWE)-corrected p = 0.05 (cluster-level) under random field theory assumptions (Kilner et al., 2005).

### Auditory Deviant-evoked ERP

To assess how attention and visual coherence affect deviant-evoked activity, the EEG data were first subject to a traditional channel-by-channel mass-univariate analysis. Epoched data were averaged over trials, separately for the deviants in At and Am and for each visual condition (Vt, Vm, Vi). Single-subject ERP data were converted into three-dimensional images (two spatial dimensions and one temporal dimension) and entered into a repeated-measures ANOVA with two within-subjects factors: attention (attended: deviant in the At stream, unattended: deviant in the Am stream) and visual coherence (coherent with the sound containing deviants: deviants in AtVt and AmVm; visual condition independent of the sound: AtVm and AmVt). The two-way repeated-measures ANOVA was implemented as a GLM in SPM12. The resulting statistical parametric maps, representing the main and interaction effects, were thresholded at p < 0.05 (two-tailed) and corrected for multiple comparisons across spatiotemporal voxels at a family-wise error (FWE)- corrected p = 0.05 (cluster-level).

In a follow-up attempt to isolate dissociable neural signatures of attention and visual coherence, we concatenated the ERP data across participants and used PCA to reduce the EEG data dimensionality and obtain spatial principal components (PCs, representing the weight of channel topographies) and temporal principal components (representing voltage time-series). The EEG data were concatenated across participants before being subjected to PCA, in order to obtain the same PCs across participants. The PCs quantified independent contributions to whole-scalp data, such that the sensitivity to those isolated components increased (relative to original data, containing a mixture of components). The first four PCs (explaining 80% of the original variance across participants) were used to extract single-participant ERP components for further analysis. Each PC was then converted into one-dimensional images (time) and subject to statistical inference using repeated-measures ANOVAs, as above. Significance thresholds were kept identical to the traditional univariate analysis, but correction for multiple comparisons was implemented across time points (rather than spatiotemporal voxels).

### Correlating timbre deviant evoked ERP magnitude with behavioral performance

Since the behavioral task was to detect deviants in the target auditory stream, we extracted the EEG responses to deviants in At and measured the peak to peak amplitude of the PCs of ERP identified above. We then calculated the Pearson correlation coefficients between the behavioral mean d’ and the mean PC amplitude over conditions (AtAmVt, AtAmVm, and AtAmVi). To reduce the number of comparisons, we limited our correlation analyses to those ERP components and factors which showed significant effects. Specifically, for the 1^st^ PC for which we have identified the significant main effect of attention (see Results), the negative and positive peaks were measured between 100 to 200 ms, and 220 to 300 ms. respectively. For the 3^rd^ PC for which we have identified significant main and interaction effects of attention and coherence (see Results), the positive and negative peaks were measured between 50 to 160 ms, and 220 to 400 ms, respectively. Prior to calculating the correlations, we fitted the behavioral performance d’ with the PC peak-to-peak amplitude using a linear regression model, and detected the outliers in each condition using Cook’s distance (threshold: 3 means of Cook’s distance).

## Results

### Behavioral results

First, we investigated whether behavioral performance was stable over time, which would warrant pooling data from all blocks. To this end, we calculated the single-participant hit rate separately for each of the 12 blocks, and fitted the data using a linear regressor representing the block number. The resulting regression coefficient (slope) was not statistically different from zero across participants (one-sample t-test, t = 1.11, p = 0.28), suggesting that there were neither significant learning nor fatigue effects during the experiment.

To investigate the effect of the visual temporal coherence on behavioral performance, we performed one-way repeated measures ANOVAs on d’ (F = 0.15, p = 0.85) (Figure 1C), hit rates (F = 0.42, p = 0.66) and false alarm rates (F = 2.12, p = 0.13). The hit rates were 69% ± 2.7%, 70% ± 2.6% and 70% ± 2.9% (mean ± SEM), and the mean false alarm rates were 4% ± 0.6%, 5% ± 0.9%, and 5% ± 1% for the three conditions (AtAmVt, AtAmVm, AtAmVi), respectively. No significant effect of visual coherence on deviant detection was observed, likely due to large variability and heterogeneity of response patterns across participants. For instance, while some participants showed behavioral benefits of visual coherence (e.g., larger d’ in AtAmVt condition than AtAmVm), others showed the opposite effects (Figure 1C). Two previous studies using similar stimulus paradigms (Atilgan & Bizley, 2021; Maddox et al., 2015) reported enhanced task performance when the target stream and visual stimulus were temporally coherent. Our failure to replicate these data may be attributable to small but perhaps important differences in the details of the experimental paradigms, especially the manipulation of visual attention (see Discussion). Furthermore, our behavioral results are consistent with the general framework of the possible effects of attention and coherence (Figure 1B), in which the relative contribution of the integration term might be small compared to the summation term. However, the aims of this study were to identify effects of auditory selective attention and AV coherence on physiological measures of neural stimulus representations, and the timbre deviants primarily served as a device for controlling and monitoring our participants’ attention. The relatively high hit rates and low false alarm rates indicate that the deviants had fulfilled that purpose.

### Stimulus reconstruction reveals temporal coherence mediated audiovisual integration

**Figure 2:**
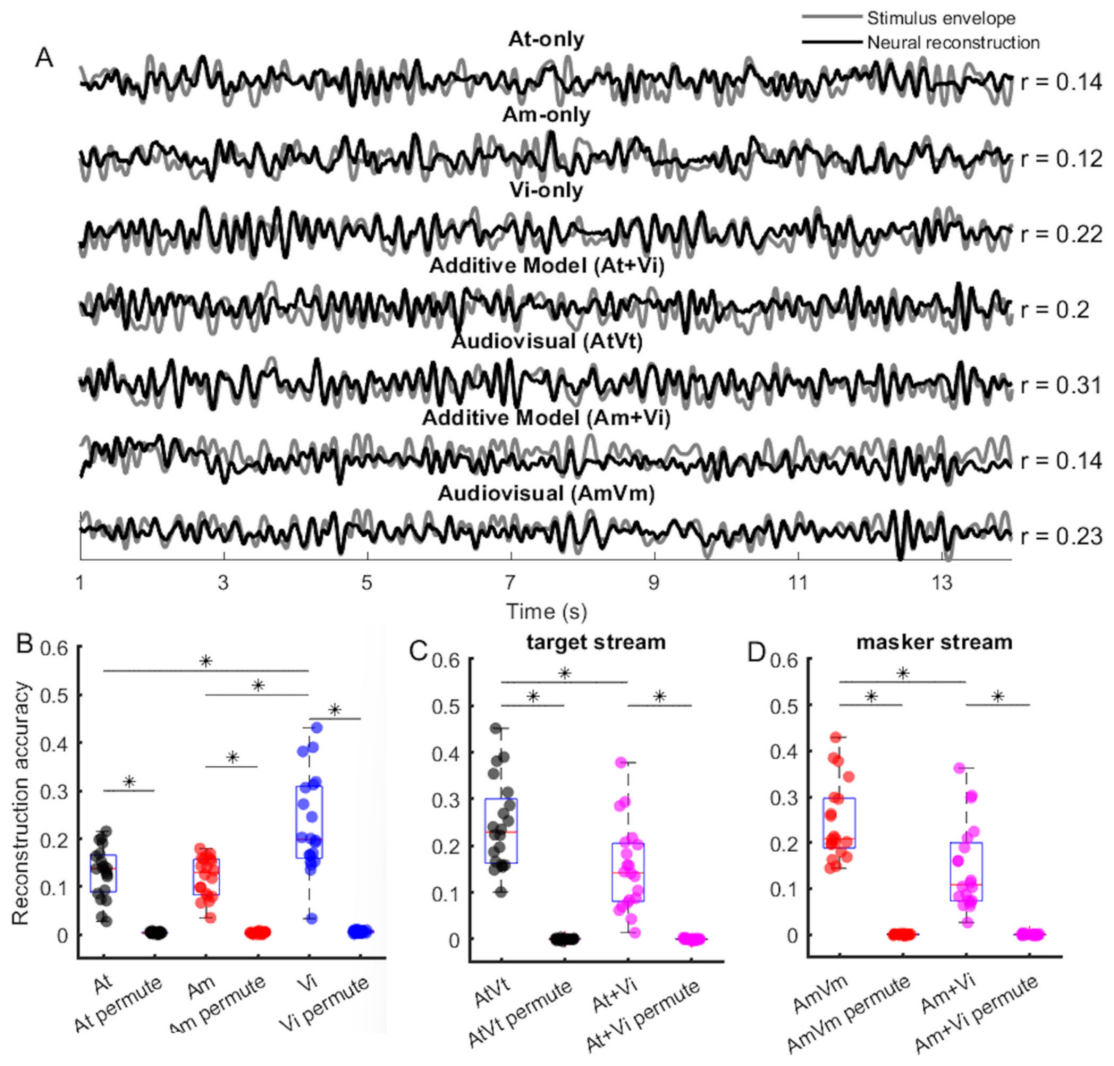
Stimulus reconstruction. **(A)** Examples of the original sound envelope (grey) with the grand-average neural reconstruction (black) overlapped. The mean reconstruction accuracy over subjects is indicated to the right. **(B)** The stimulus reconstruction accuracy for each stream in the independent condition AtAmVi was significantly better than chance (permutation test). Each dot represents one subject. **(C, D)** The stimulus reconstruction accuracy using the AV decoder and A+V decoder for the target and masker sound was significantly better than chance (permutation test), respectively.

To investigate the occurrence of AV integration at both attended and unattended conditions, we reconstructed an estimation of the sound envelope from the recorded EEG waveforms. We used the condition in which the visual stimulus was independent of both auditory streams to estimate unimodal reconstructions for the target auditory stream (At), the masker stream (Am) and the visual stream (Vi) (Figure 2B). From this condition we could independently estimate unisensory response elements, without introducing some of the confounds inherent in comparing activity across multisensory and unisensory trials, where prestimulus expectation and attention may differ (Mishra et al., 2007, Rohe et al. 2019). We first confirmed that the unimodal reconstructions for all conditions were significantly better than the chance estimated using a permutation test. From the unisensory reconstructions, we estimated the response to stimuli in which the visual stimulus was coherent with one or the other stream by linear summation. This linear summation model was compared to an integration model in which audiovisual envelopes were reconstructed based on the responses to conditions in which the visual stimulus was temporally coherent with one or the other stream (i.e., AtVt and AmVm) (Figure 2A). Testing for the interaction of attention and integration in a two-way repeated measures ANOVA, we only found that the main effect of integration was significant (F = 491.8, p <0.001). In post-hoc comparisons, we observed that the average reconstruction accuracy of the AV decoder was significantly higher than that of the A+V decoder for both the target stream (Figure 2C, Wilcoxon signed-rank test p < 0.001) and the masker stream (Figure 2D, Wilcoxon signed-rank test p < 0.001), consistent with AV integration occurring independently of attention.

### Forward models highlight attentional modulation of auditory responses

We next asked how temporal coherence and attention affect AV integration across the different EEG channels by estimating temporal response functions (TRFs) of each channel. While stimulus reconstruction predicts the accuracy of cortical tracking of the amplitude envelope by using multichannel EEG response (and may therefore be dominated by visual responses), TRFs reflect the linear transformation of the sound envelope to the neural responses at each EEG channel. We first explored whether we could observe similar evidence of audiovisual integration from the TRF estimations as we did with the stimulus reconstruction. We estimated unisensory TRFs for the auditory target stream (TRF_At_), the auditory masker stream (TRF_Am_), and the visual stimulus (TRF_Vi_), separately, from the response in the condition AtAmVi, in which temporal envelopes of all three streams were independent. We then estimated the TRF_AtVt_ and TRF_AmVm_ using the responses in the condition AtAmVt and AtAmVm, respectively.

**Figure 3:**
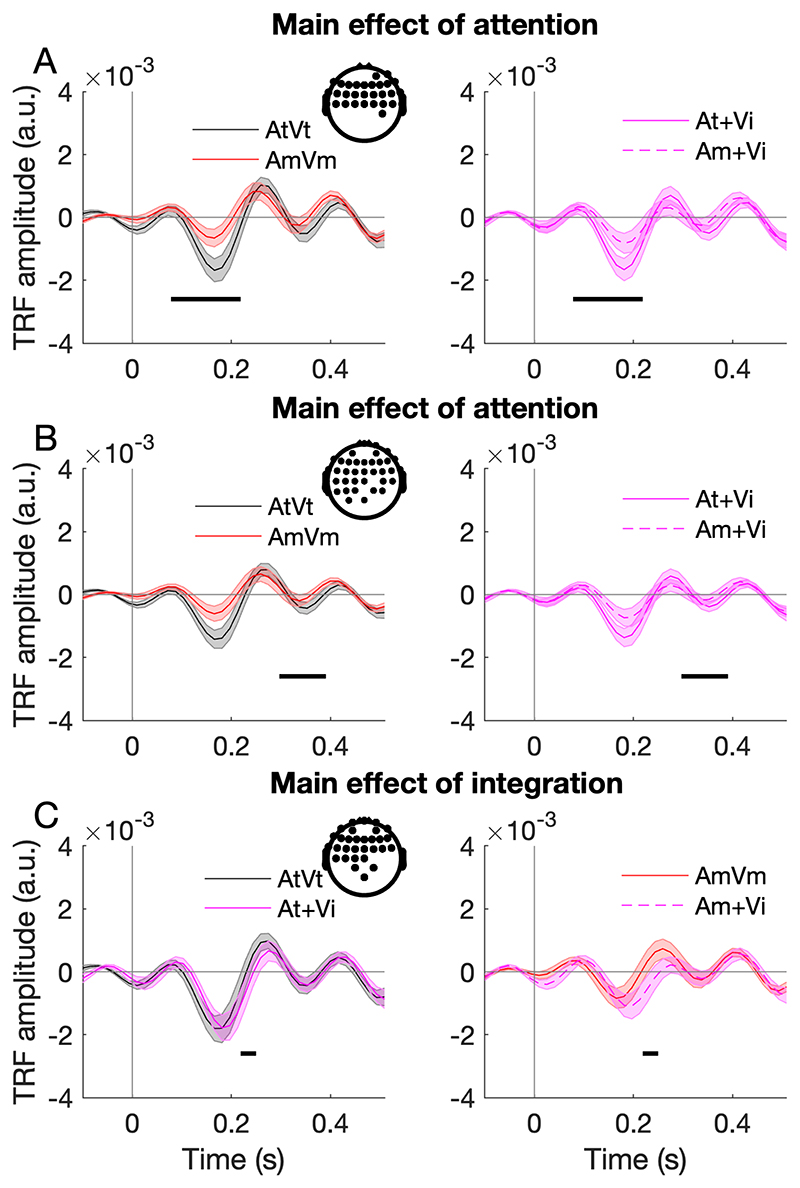
Temporal response function analysis. **(A, B)** Left panel, the TRF estimated for coherent target stream (AtVt) had a stronger amplitude than that for the masker stream (AmVm). Right panel, the summation of TRFs estimated for the target stream (At + Vi) was significantly stronger than that for the masker stream (Am + Vi). **(C)** Left panel, for the target sound (At) condition, the TRF estimated for coherent AV streams (AtVt) was not significantly different from the summation of TRFs estimated for independent AV streams (At + Vi). Right panel, for the masker sound (Am) condition, the TRF estimated for coherent AV streams (AmVm) had a stronger amplitude than the summation of TRFs estimated for independent AV streams (Am + Vi). Shaded areas indicate SEM (standard error of the mean) across subjects. The topographical plot shows the EEG channel locations with a significant difference. Black horizontal bars: p_FWE_ < 0.05 (based on the main effects in the ANOVA; see Results).

To investigate how the cortical representation of amplitude envelopes was influenced by attention and AV integration, we used a two-way repeated measures ANOVA to assess the influence of AV integration and attention on the TRF amplitudes across all EEG channels. We observed a significant main effect of attention (Figure 3A anterior cluster, 78 ms to 219 ms, F_max_ = 26.68, Z_max_ = 4.51, p_FWE_ < 0.001; Figure 3B anterior and central cluster, 297 to 391 ms, F_max_ = 27.98, Z_max_ = 4.61, p_FWE_ = 0.008) and integration (Figure 3C anterior cluster, 219 to 250 ms, F_max_ = 14.12, Z_max_ = 3.35, p_FWE_ = 0.009).

In summary, we observed evidence that AV integration occurred both in the target and masker auditory stream when measures of stimulus reconstruction accuracy were used to analyse the neural responses to the sound envelopes. Analysis of TRFs amplitude across all EEG channels showed that attention modulated the magnitude of the TRF. AV integration was observed for the masker stream in central and frontal channels. The attention effect was observed for a subset of channels in the TRF analysis but not in the stimulus reconstruction, which utilized the responses across all channels. The other possible reason that attentional effects were observed with the TRF and not the stimulus reconstruction, is that the latter might be dominated by the responses to visual stimulus (Figure 2B). Taken together, our results suggest audio-visual integration occurs automatically, prior to attentional modulation.

### Effects of audiovisual temporal coherence and selective attention on deviant-evoked responses

The analysis so far has focused on the neural responses to the amplitude envelopes of the audiovisual scene, and has revealed evidence for both attentional modulation of acoustic responses, and AV integration of temporally coherent cross-modal sources. Since, in the temporally coherent conditions, the visual and auditory streams convey redundant information, this integration falls short of reaching the stricter definition of binding proposed by Bizley et al., (2016) which requires an enhancement of independent features that are not those which link the stimuli across modalities. Here, the presence or timing of the auditory timbre deviants that listeners detected in the selective attention task are not predicted by the amplitude changes of the audio or visual envelopes, and they thus provide a substrate with which to explore binding.

**Figure 4:**
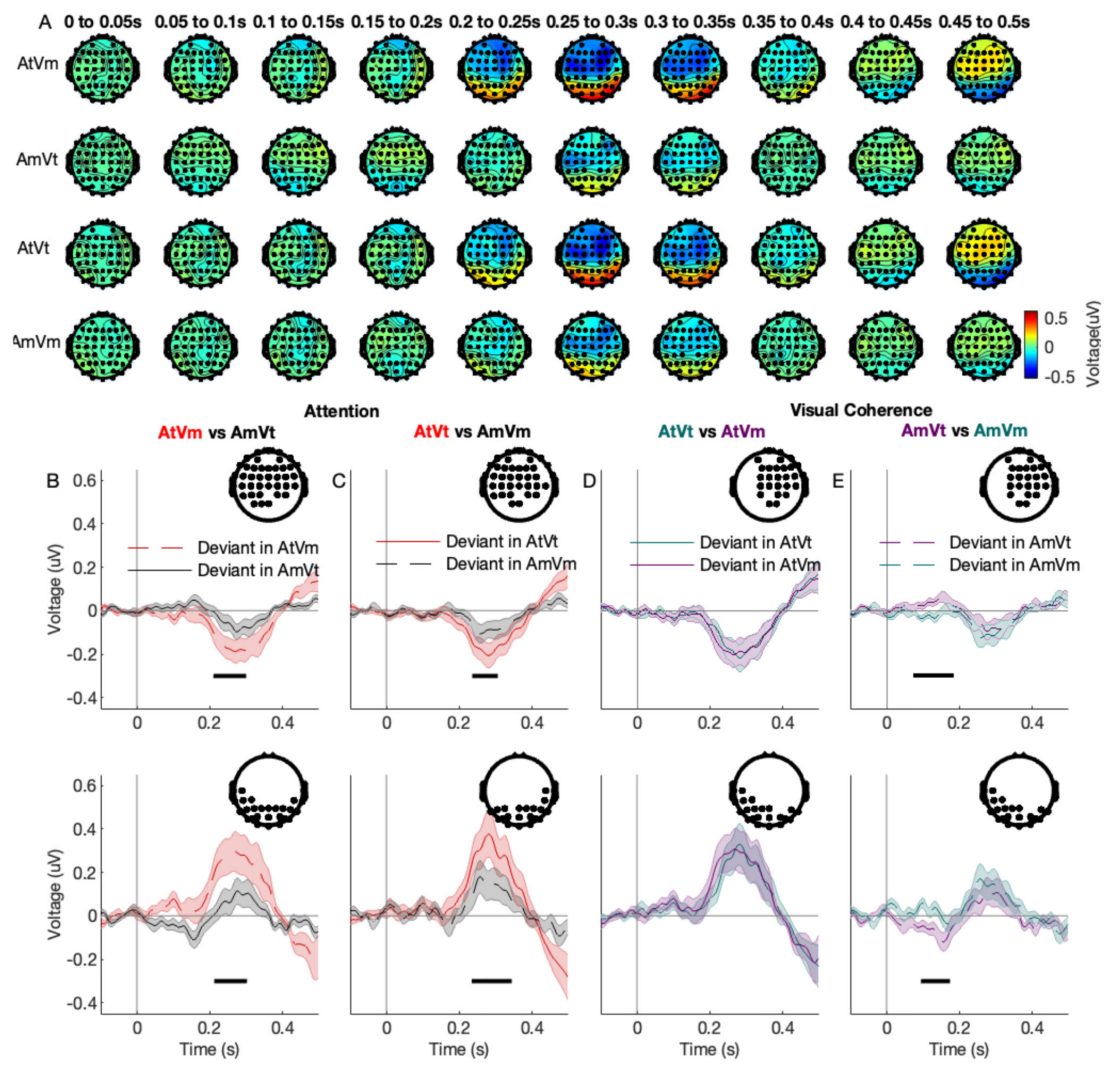
Grand-average deviant-evoked ERPs over participants and channels across conditions. **(A)** Scalp topographies from deviant onset to 0.5 s after onset. Each row represents one condition (from top to bottom, the condition corresponds to AtVm, AmVt, AtVt, and AmVm, respectively), each column represents one 50-ms time window. **(B)** Deviants presented in the incoherent target stream (red dashed lines) and masker stream (grey solid lines); **(C)** Deviants presented in the AV coherent target (red solid lines) and masker stream (black dashed lines); **(D,E)** Deviants presented in the target and masker stream in each of the two attentional conditions (left: masker stream; right: target stream); The topographical plots in panels (A-C) show the EEG channel locations where a significant ERP amplitude difference between the two conditions (as indicated at the top of each plot) was observed (FWE-corrected) except. The topographical plot in (D) shows the EEG channel locations same as the locations in (C). The black bar represents the time segment with a significant difference between the deviants in two different conditions. Shaded areas represent SEM across subjects.

To investigate how AV temporal coherence and attention affect the deviant-evoked responses, we compared the ERPs evoked by deviants embedded in At and Am streams, and, in order to look for evidence of binding, asked how audiovisual temporal coherence modulated these responses (Figure 4). As shown in scalp topographies which visualize the response change over time for each condition (Figure 4A), the deviant-evoked response in the target stream was clearly stronger than that in the masker stream.

Accordingly, in a traditional channel-by-channel mass-univariate analysis, correcting for multiple comparisons across all channels and time points, we observed a significant main effect of attention (anterior cluster, 196 to 302 ms, F_max_ = 16.87, Z_max_ = 3.65, p_FWE_ < 0.001; posterior cluster, 210 to 320 ms, F_max_ = 21.32, Z_max_ = 4.08, p_FWE_ < 0.001) and a significant interaction effect of attention and temporal coherence (anterior cluster, 62 to 146 ms, F_max_ = 11.26, Z_max_ = 2.99, p_FWE_ = 0.036, posterior cluster, 58 to 146 ms, F_max_ = 16.92, Z_max_ = 3.66, p_FWE_ = 0.03 ). No main effect of temporal coherence was observed.

Significant post-hoc comparisons between conditions were consistent with the main effect of attention: for both temporally coherent and temporally independent streams, the deviant response in the target always exceeded that of the masker. The amplitude of the ERP evoked by timbre deviants presented in the target stream (AtVm) was significantly larger than that in the masker stream (AmVt) in two clusters: negative peak enhancement was observed over anterior channels (Figure 4B the first row, 210-300 ms after deviant onset, p_FWE_ < 0.001, T_max_ = 4.23), and positive peak enhancement over posterior channels (Figure 4B the second row, 212-302 ms after deviant onset, p_FWE_ < 0.001, T_max_ = 4.68). In the AV coherent stream, we observed that ERP amplitude evoked by the timbre deviants in the attended coherent stream (AtVt) was significantly stronger than in the unattended coherent stream (AmVm) in two clusters: one over the central and frontal channels between time lag 236 to 310 ms (Figure 4C the first row, cluster level p_FWE_ < 0.001, T_max_ = 3.8), and one over posterior channels between time lag 234 to 350 ms (Figure 4C the second row, cluster level p_FWE_ = 0.007, T_max_ = 4.18).

Post-hoc comparisons also allowed us to examine the interaction between temporal coherence and attentional condition. We observed that the amplitude of ERP evoked by deviants in the masker stream was significantly smaller when this was accompanied by a temporally coherent visual stimulus (Figure 4E). The deviant induced ERP was smaller in the AmVm condition than in the AmVt condition in two clusters: one over the central and frontal channels between time lag 74 to 186 ms (Figure 4E the first row, cluster level p_FWE_ = 0.011, T_max_ = 4.38), and one over left temporal and posterior channels between time lag 94 to 180 ms (Figure 4E the second row, cluster level p_FWE_ = 0.005, T_max_ = 3.72). In contrast, audiovisual temporal coherence did not influence the size of the deviant response in the target stream (Figure 4D).

**Figure 5:**
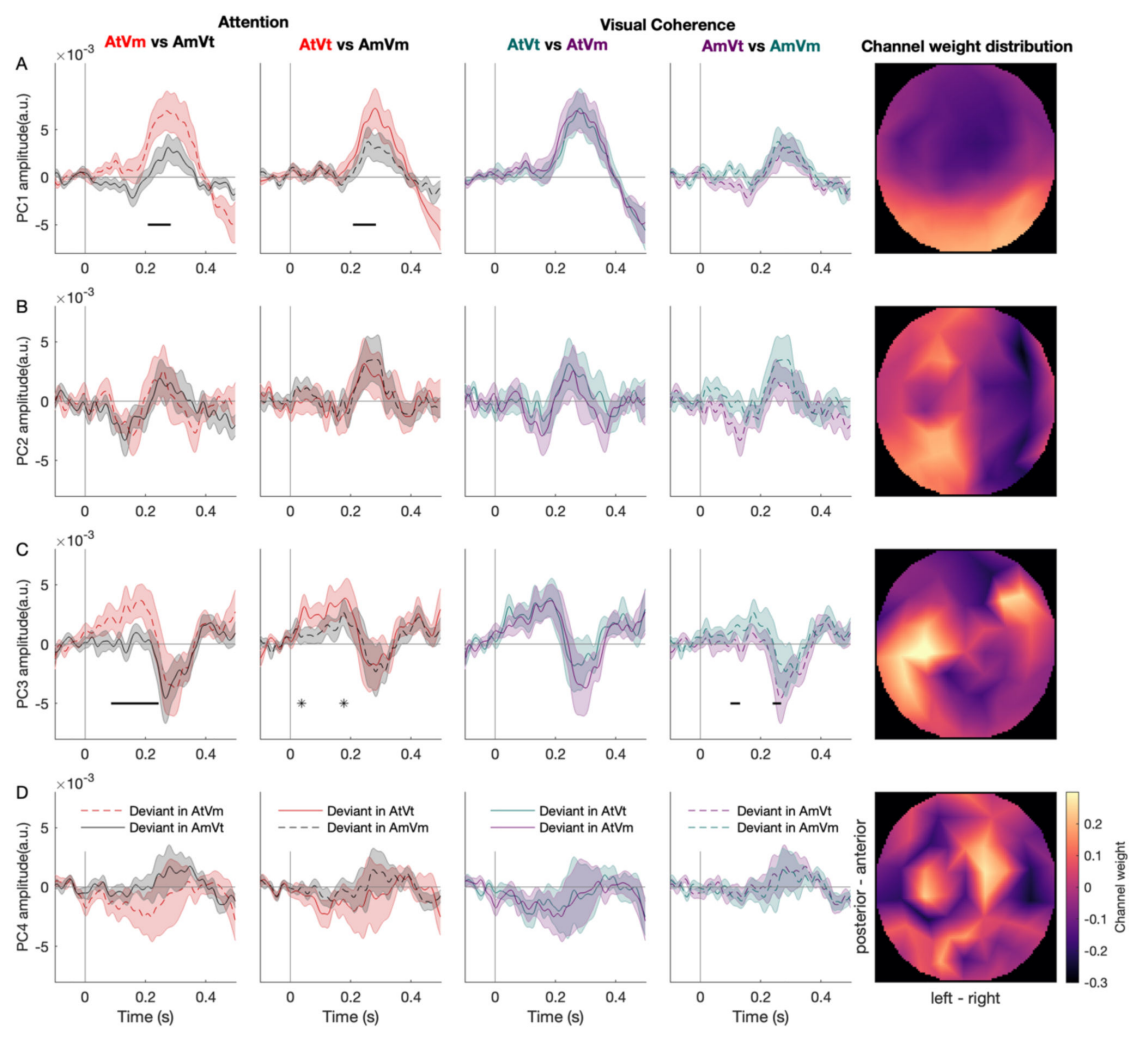
Attentional enhancement of deviant-evoked ERPs: principal component analysis. (A,B,C,D) Deviant-evoked response for the 1^st^ PC, 2^nd^ PC, 3^rd^ PC, and 4^th^ PC of ERP, respectively. The first two columns represent the attention effect on the AV incoherent conditions (AtVm in red dashed lines and AmVt in black solid lines) and AV incoherent conditions (AtVt in red solid lines and AmVm in black dashed lines). Black bars indicate the time periods with a significant difference between the two conditions, and black asterisks indicate the time points with a significant difference between the two conditions. Shaded areas indicate SEM across subjects. The third and fourth columns represent the AV coherence effect on the target conditions (AtVt in blue solid lines and AtVm in purple solid lines) and masker conditions (AmVt in purple dashed lines and AmVm in blue dashed lines). The fifth column represents the spatial topography map of the principal component weights across channels. Color indicates the weight (warm: high, cool: low).

From the mass-univariate ERP data analysis (i.e., when analysing all channels and correcting for multiple comparisons across channels and time points), attention was the main modulator of the size of the deviant response, with temporal coherence only influencing the deviant responses in the masker stream. In a follow-up exploratory analysis, we investigated whether effects of visual coherence, as well as attention, can be identified when EEG channels are grouped into principal spatiotemporal components explaining different sources of variance. To this end, we performed a principal component analysis to extract the spatiotemporal components of the ERP, and performed separate two-way repeated measures of ANOVAs with two main factors: attention (attended and unattended) and visual coherence (coherent and incoherent), on the first four principal components (PCs) in the time domain. These four PCs together explained 80% of the original variance. The analysis of the 1^st^ PC (Figure 5A, explaining 67% of the original variance) only showed a main effect of attention (time lag between 208 to 284 ms, F_max_ = 32.53, Z_max_ = 4.92, p_FWE_ < 0.001). No main or interaction effects were found to be significant for the 2^nd^ and 4^th^ PC (Figure 5B and Figure 5D, explaining 6% and 3% of the original variance, respectively). However, the analysis of the 3^rd^ PC (explaining 4% of the original variance) showed a main effect of attention (time lag between 8 to 84 ms, F_max_ = 43.33, Z_max_ = 5.53, p_FWE_ < 0.001; 134 to 170 ms, F_max_ = 77.98, Z_max_ = 6.88, p_FWE_ < 0.001; 260 ms, F_max_ = 26.54, Z_max_ = 4.50, p_FWE_ < 0.001), coherence (time lag at 346 ms, F_max_ = 9.98, Z_max_ = 2.81, p_FWE_ < 0.001), and the interaction effect between attention and visual coherence (time lag between 214 to 238 ms, F_max_ = 14.82, Z_max_ = 3.43, p_FWE_ < 0.001). We therefore subjected the 3^rd^ PC to further analyses described below.

Post-hoc tests on this principal component supported the idea that attention dominates the neural response, but that temporal coherence can modulate it. In keeping with the main ERP results, the main effect of audiovisual temporal coherence was apparent in the unattended stream, suggesting that the effect of attention may be strong enough to elicit a ceiling effect. Specifically, we observed main effect of attention (AtVm > AmVt: 86 - 244 ms, cluster-level p_FWE_ < 0.001, T_max_ = 8.16; AtVt > AtVm at 38 ms, cluster-level p_FWE_ < 0.001, T_max_ = 4.60; at 178 ms, cluster-level p_FWE_ = 0.032, T_max_ = 3.45; Figure 5C). The effect of attention on the incoherent stream extends over more time points than the effect of attention on the coherent stream. Consistent with this being due to a temporal coherence mediated enhancement of the masker stream, the deviant-evoked responses in the masker were significantly greater when accompanied by a temporally coherent visual stimulus (AmVm>AmVt: 100-132 ms, T_max_ = 3.79, cluster-level p_FWE_ < 0.001; 240 to 268 ms, cluster-level p_FWE_ < 0.001, T_max_ = 3.55; Figure 5C). The PC was dominated by the responses from the left temporal and right frontal channels (Figure 5C, last column).

### Correlations between behavioral performance and EEG

**Figure 6:**
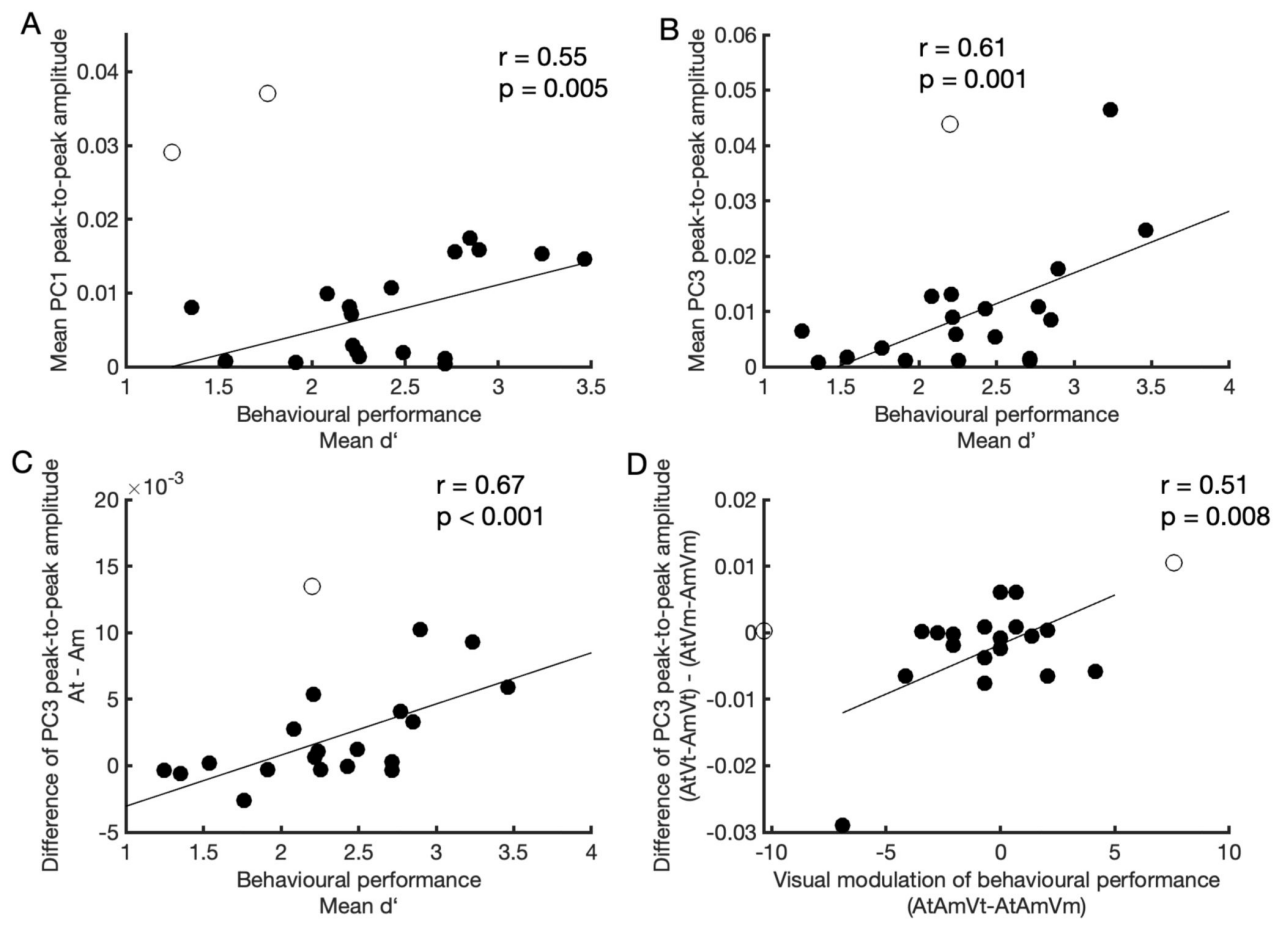
Correlations between the behavioral performance and EEG responses. **(A, B)** The correlation between mean d’ and the mean 1^st^ PC and 3^rd^ PC peak-to-peak amplitude over conditions (AtAmVt, AtAmVm, and AtAmVi), respectively. **(C)** The correlation between mean d’ and the mean 3^rd^ PC peak-to-peak amplitude (At - Am). **(D)** Visual coherence modulation of behaviour performance with EEG responses. The correlation between the hit rate difference (AtAmVt - AtAmVm) and the 3^rd^ PC peak-to-peak amplitude (AtVt – AmVt vs AtVm - AmVm). The unfilled circles represent outliers. (P value corrected for multiple comparison.)

To examine the relationship between the EEG responses and behavioral performance, we calculated Pearson correlation coefficients between measures of behavioural performance and neural activity. Outliers were deleted using Cook’s distance if the distance was larger than 3 times the means of Cook’s distance. We first considered whether the magnitude of the deviant response in the target stream correlated with overall behavioural performance (mean d’ across all visual conditions), reasoning that participants with a stronger deviant response might be better able to accurately report timbre deviants. For both PC1 and PC3, the peak-to-peak PCs of ERP amplitudes obtained for the deviants in the target stream (At) correlated with overall d’ performance (PC1 peak-to-peak amplitude: Figure 6A, r = 0.55, p = 0.019; PC3: Figure 6B, r = 0.61, p = 0.005).

The auditory selective attention task required that participants not only detect timbre deviants, but that they successfully differentiated target and masker events. We therefore hypothesised that listeners who more successfully engaged selective attention mechanisms might show larger differences in the magnitude of deviant response to target and masker deviants. To test this we subtracted the peak to peak amplitude of EEG responses for masker deviants from the peak to peak amplitude to target deviants, and then measured the correlation between the EEG responses difference with the behavioral performance (d’). This relationship was observed for PC3 (Figure 6C, r=0.67, p=0.001), but not PC1 (r=-0.01, p=0.971)..

Finally, while the visual condition did not significantly influence behavioural performance at the group level, there was significant heterogeneity within our listeners. To determine whether modulation of behavioural performance by the visual stimulus correlated with the magnitude of the attention × visual condition interaction in PC3, we considered the difference in the normalised d’ performance for target-coherent and masker-coherent trials (i.e. the difference in target-coherent d’ and masker-coherent performance d’ / overall d’) and correlated this with the difference in the attentional modulation of the 3^rd^ PC peak-to-peak amplitude across visual conditions, i.e. AtVt-AmVt vs AtVm-AmVm (Figure 6D, r = 0.51, p = 0.031). While the correlation was significant and in the predicted direction (i.e. participants who showed a benefit for target-coherent trials had a greater attentional modulation in the target-coherent condition), we note that it’s principally driven by a single participant whose removal renders the correlation non-significant.

## Discussion

This study used an auditory selective attention task, performed in the presence of a temporally modulated visual stimulus, to dissect the neural signatures of selective attention and audiovisual temporal coherence. Our EEG data of envelope responses reveal evidence for audiovisual integration of temporally coherent audiovisual envelopes which occurred independently of selective attention. Meanwhile, selective attention had a strong effect on the amplitude of TRFs derived from the envelope responses, with TRFs corresponding to target streams yielding higher amplitudes than those corresponding to masker streams. To further investigate audiovisual binding we examined the EEG responses to the timbre deviants which occurred independently of the amplitude envelopes of the audio(visual) streams. The fact that the EEG responses elicited by the timbre deviants were affected by the visual coherence of the stimulus can be interpreted as evidence that temporal coherence in the audiovisual streams favored the emergence of a fused audiovisual percept, which contrasts more strongly against the deviants than a purely auditory stream would. In direct support of this notion, we observed that, in some spatiotemporal components of the neural response, audiovisual temporal coherence interacted with selective attention.

### Temporal coherence based AV integration occurs independently of attention

Based on the stimulus envelope reconstruction analysis, we found that the cortical responses to the AV amplitude envelope were better explained by an AV integration model than by a linear summation (A+V) model in both the attended and unattended streams, suggesting attention was not required to link audio and visual streams. Our study thus provides evidence that AV integration based on temporal coherence between the auditory and visual stream can occur independently of attention. This result is in contrast to previous studies using speech as stimuli. Ahmed et al., (2021) found AV integration was only observed for attended speech stream, demonstrating that responses to attended speech were better explained by an AV model, while the responses to unattended speech were better explained by the A+V model. However, their integration model outperformed the linear summation model for unattended speech at very short (0-100 ms lag) latencies, suggesting that distinct multisensory computations occur at different processing stages. In contrast to studies utilizing natural speech and videos of faces, our visual disc was much simpler. One possibility, which is already noted in Atilgan et al. (2018), is that bottom up audiovisual integration does occur independently of attention for simple non-speech stimuli. Another possibility is that watching a competing talker is more distracting than watching an uninformative disc, perhaps leading to observers actively suppressing a competing face in the context of a selective attention task. A final difference might be that subjects in Ahmed et al. (2021) were instructed to look at the eyes of the face, whereas our listeners fixated on the disc itself; potentially the radius changes of the disk, presented at the fovea, provide a more salient temporal cue. In support of this possibility, we note that the stimulus reconstruction accuracy of the visual-only decoder in the independent condition was quite high, and significantly larger than that of the audio-only decoder.

We used a forward model to examine the cortical representation of the sound amplitude envelope across all EEG channels. Two-way repeated measures ANOVA indicated significant main effects of attention and integration. In the unattended sound stream, the TRF_AV_ amplitude was significantly stronger than the summation of TRF_A_ and TRF_V_ amplitude, which suggests that AV integration occurs independently of attention. This result is consistent with our results from the envelope reconstruction (Figure 2), as well as a previous study from Crosse et al (2015), both in terms of the direction of the effect (AV vs. A+V) and its latency in the ~200 ms range. Furthermore, attention strongly modulated the TRF, with the TRF_AV_ amplitude for the target stream being significantly larger than that for the masker stream. This finding is consistent with previous studies, demonstrating an enhancement of attended speech streams (Ding & Simon, 2012; Mesgarani & Chang, 2012) and audiovisual streams (Zion Golumbic et al., 2013). An open question is why audiovisual temporal coherence did not influence the attended stream TRF_AV_. Perhaps the enhancement of the TRF by attention generated a ceiling effect, or possibly if we had required subjects to attend to the visual stimulus we might have observed stronger audiovisual interactions. Nevertheless, our TRF results reveal the effects of both audiovisual temporal coherence and attention on the TRF amplitude.

### Attention and coherence effects on the deviant evoked responses

In this study, we adapted the behavioral paradigm of previous studies (Atilgan & Bizley, 2021; Maddox et al., 2015), however, we failed to replicate the behavioural findings. Two key differences may explain this: first, the magnitude of the timbre deviants was increased, which effectively rendered the task easier. The overall d’ scores are higher in the current dataset than in previous ones. A recent study (Cappelloni et al., 2022) also suggested that the temporal coherence of the visual stream might not provide additional benefit if the two auditory streams were easily segregated. Second, in these previous studies, listeners were also required to detect occasional colour deviants in the visual stimulus, which required them to maintain some level of attention towards the visual modality. In our experiment, the visual stimulus neither contained deviants of its own, nor did it provide cues that might facilitate the detection of auditory deviants. Within the framework of the model included in Figure 1B, attending to the visual stream would lead to further enhancement. It is possible that this difference explains why, at the group level, we did not observe a significant effect of audiovisual temporal coherence on auditory deviant detection.

A whole-scalp analysis of deviant-evoked ERPs brought evidence for a main effect of attention, with the latency of the effect corresponding to a P300 time window. The P300 is a later component in response to novelty occurring between 200-600 ms relative to deviant onset, and has been previously described for the auditory and visual modalities (Friedman et al., 2001). Previous studies showed that the P300 is attention-dependent (Polich et al., 2007), consistent with our findings. The anterior-posterior topography of the effect shown on Figure 4 is due to our choice of re-referencing to the average of all channels. In addition to this robust modulation of the deviant response by attention, a further PCA based on the timbre deviant elicited ERPs revealed interactions between attention and audiovisual temporal coherence. For specific principal components, there was an attention-dependent enhancement of the deviant-evoked responses in the target stream independent of the visual coherent. This suggests that the attentional modulation of the target stream is sufficiently strong that temporal coherence exerts no additional effect. We found the main effect of attention to modulate activity at very early latencies (8 - 84 ms), although cluster-based statistics do not indicate that all time points within this time window show significant effects, but rather that there are some time points within the cluster that show significant effects. The post-hoc test showed that the early peak of the attention effect was at 38 ms (Figure 5C, AtVt vs AtVm). Previous studies has shown similarly early attention effects on auditory responses, e.g. in a previous MEG study (Auksztulewicz et al., 2015), a main effect of attention was observed around 27-40 ms after tone onset. Such early latencies are consistent with earlier results obtained in attentional paradigms based on auditory filtering (Rif et al., 1991) and could be interpreted as evidence of attentional gating (Lange 2013). However, for the unattended stream, temporal coherence does enhance the deviant evoked response in the masker stream. One possibility therefore is that in this paradigm the attentional modulation was sufficiently strong that, for target sounds, there was a ceiling effect preventing any further modulation by audiovisual temporal coherence (equivalent in the model in Figure 1B to the magnitude of attentional enhancement rendering small changes due to audiovisual integration as irrelevant to the eventual summed activity). Some caution is required in interpreting these results given that the 3^rd^ PC accounted for only 4% of the variance in the EEG data, but it is noteworthy that this PC also correlated with differences in task performance. The magnitude of attentional modulation scaling with overall behavioural performance d’ (Figure 6C). There was some evidence for a correlation between the extent to which the visual condition influenced behavioural performance and the magnitude of the temporal coherence dependent attentional effects (Figure 6D), although this requires replication, preferably in the context of task parameters that more reliably elicit a modulation of task performance by audiovisual temporal coherence. That we see significant audiovisual integration in the envelope tracking responses, but not in behaviour or in the main ERP analysis (Figure 4) of the timbre deviants, potentially suggests that both behaviour and timbre deviant responses are dominated by attentional effects. Future experiments could make attentional selection harder, for example by making the pitch or timbre of the two streams more similar, in order to determine whether it is possible to unmask audiovisual temporal coherence effects that are hinted at by our PCA of the timbre deviant responses.

Our results are consistent with previous studies on ‘cocktail party effect’ speech stream segregation, in which congruent visual stimuli enhanced the cortical representation of the speech envelope of attended speech streams relative to unattended streams (Crosse, Di Liberto, & Lalor, 2016; Golumbic et al., 2013). However, unlike in these previous studies, where visual speech provided temporal and contextual information about the auditory envelope, we used a simple disc as a visual stimulus, which provided no information about the auditory deviant. While previous studies have demonstrated that attention dedicated to one feature of an object may enhance the responses to other features of the object in both auditory (Alain & Arnott, 2000; Maddox & Shinn-Cunningham, 2012; Shamma et al., 2011; Shinn-Cunningham, 2008) and visual modalities (Blaser et al., 2000; O’Craven et al., 1999), our results provide new evidence that temporal coherence modulates the attentional enhancement of the neural response to the timbre deviants (“other” features) of the AV object.

In summary, we examined the temporal coherence and attention effect on neural responses to the continuous sound envelope and the deviant evoked response, respectively. Temporal coherence facilitated the audiovisual integration independent of attention, and attention further enhanced the audiovisual integration of the coherent audiovisual stream. Attention amplified a large portion of the deviant-evoked response independent of temporal coherence, while coherence only modulated deviant-evoked responses in the unattended auditory stream. These results provide evidence for partly dissociable neural signatures of bottom-up (coherence) and top-down (attention) effects in AV object formation.

## Significance Statement

Temporal coherence between auditory stimuli and task-irrelevant visual stimuli can enhance behavioral performance in auditory selective attention tasks. However, how audiovisual temporal coherence and attention interact at the neural level has not been established. Here, we measured EEG during a behavioral task designed to independently manipulate AV coherence and auditory selective attention. While some auditory features (sound envelope) could be coherent with visual stimuli, other features (timbre) were independent of visual stimuli. We find that audiovisual integration can be observed independently of attention for sound envelopes temporally coherent with visual stimuli, while the neural responses to unexpected timbre changes are most strongly modulated by attention. Our results provide evidence for dissociable neural mechanisms of bottom-up (coherence) and top-down (attention) effects on AV object formation.
